# Serum Polyfluoroalkyl Concentrations, Asthma Outcomes, and Immunological Markers in a Case–Control Study of Taiwanese Children

**DOI:** 10.1289/ehp.1205351

**Published:** 2013-01-08

**Authors:** Guang-Hui Dong, Kuan-Yen Tung, Ching-Hui Tsai, Miao-Miao Liu, Da Wang, Wei Liu, Yi-He Jin, Wu-Shiun Hsieh, Yungling Leo Lee, Pau-Chung Chen

**Affiliations:** 1 Department of Biostatistics and Epidemiology, and; 2 Department of Occupational and Environmental Health, School of Public Health, China Medical University, Shenyang, China; 3 Department of Epidemiology, School of Public Health, Saint Louis University, St. Louis, Missouri, USA; 4 Institute of Epidemiology and Preventive Medicine, College of Public Health, National Taiwan University, Taipei, Taiwan; 5 School of Environmental Science and Technology, Dalian University of Technology, Dalian, China; 6 Department of Pediatrics, National Taiwan University Hospital, Taipei, Taiwan; 7 Department of Public Health, College of Public Health, National Taiwan University, Taipei, Taiwan; 8 Institute of Biomedical Sciences, Academia Sinica, Taipei, Taiwan; 9 Institute of Occupational Medicine and Industrial Hygiene, College of Public Health, National Taiwan University, Taipei, Taiwan; 10 Department of Environmental and Occupational Medicine, National Taiwan University College of Medicine and National Taiwan University Hospital, Taipei, Taiwan

**Keywords:** asthma, AEC, ECP, IgE, perﬂuorinated compounds

## Abstract

Background: Perﬂuorinated compounds (PFCs) are ubiquitous pollutants. Experimental data suggest that they may be associated with adverse health outcomes, including asthma. However, there is little supporting epidemiological evidence.

Methods: A total of 231 asthmatic children and 225 nonasthmatic controls, all from northern Taiwan, were recruited in the Genetic and Biomarkers study for Childhood Asthma. Structure questionnaires were administered by face-to-face interview. Serum concentrations of 11 PFCs and levels of immunological markers were also measured. Associations of PFC quartiles with concentrations of immunological markers and asthma outcomes were estimated using multivariable regression models.

Results: Nine PFCs were detectable in most children (≥ 84.4%), of which perfluorooctane sulfonate (PFOS) was the most abundant (median serum concentrations of 33.9 ng/mL in asthmatics and 28.9 ng/mL in controls). Adjusted odds ratios for asthma among those with the highest versus lowest quartile of PFC exposure ranged from 1.81 (95% CI: 1.02, 3.23) for the perfluorododecanoic acid (PFDoA) to 4.05 (95% CI: 2.21, 7.42) for perfluorooctanic acid (PFOA). PFOS, PFOA, and subsets of the other PFCs were positively associated with serum IgE concentrations, absolute eosinophil counts (AEC), eosinophilic cationic protein (ECP) concentrations, and asthma severity scores among asthmatics.

Conclusions: This study suggests an association between PFC exposure and juvenile asthma. Because of widespread exposure to these chemicals, these findings may be of potential public health concern.

Perfluorinated compounds (PFCs) include a class of human-made organic chemicals composed of a fluorinated carbon backbone of varying length, terminated by a carboxylate or sulfonate functional group. Such PFCs are extremely stable, thermally, biologically, and chemically, and additionally possess hydrophobic and lipophobic characteristics that enable products coated in them to repel both oil and water and resist staining (Conder et al. 2008; Hoffman et al. 2010). Accordingly, PFCs are widely used, for example in surfactants, emulsifiers, food packaging, nonstick pan coatings, fire-fighting foams, paper and textile coatings, and personal care products (Lau et al. 2007; Lindstrom et al. 2011; Renner 2001).

This combination of extreme resistance to degradation and environmental ubiquity has raised concerns in recent years (Giesy and Kannan 2001; Lau et al. 2007). Furthermore, studies have shown that PFCs accumulate among the higher trophic level of the food chain, such as predators and human beings (Conder et al. 2008; Houde et al. 2006; Noorlander et al. 2011). Although data from the National Health and Nutrition Examination Survey have indicated a decrease in serum PFC concentrations in the general U.S. population since the production of some PFCs has been phased out [for example, the average concentration of perfluorooctane sulfonate (PFOS) decreased from 30.4 ng/mL in 1999 to 13.2 ng/mL in 2008] (Kato et al. 2011), PFCs are still manufactured abroad (Paul et al. 2009). PFCs bioaccumulate by binding to proteins in the liver and serum, in contrast with many other persistent organic pollutants that persist primarily in adipose tissue (Conder et al. 2008), and they are slowly eliminated without biotransformation (Lau et al. 2007). Serum half-life estimates in an occupationally exposed cohort ranged from 5.4 years for PFOS to 8.5 years for perfluorohexane sulfonic acid (PFHxS) (Olsen et al. 2007).

Several attempts have been made to understand the toxicological hazards that may be associated with exposure. Early animal studies focused almost exclusively on exposure to PFOS and perfluorooctanic acid (PFOA), two of the most common PFCs. Evidence of several potential effects has been reported based on experimental studies, including hepatotoxicity, immunotoxicity, developmental toxicity, reproductive toxicity, neurotoxicity, endocrine toxicity, and tumors of the liver, thyroid, and mammary glands (Corsini et al. 2011; Lau et al. 2004, 2007; Olsen et al. 2009).

Preliminary data suggest that PFCs have the potential to exacerbate atopic diseases such as asthma. In a murine model of asthma, Fairley et al. (2007) found that PFOA increased serum levels of immunoglobulin E (IgE) and enhanced the hypersensitivity response to ovalbumin, suggesting that PFOA exposure may augment the IgE response to environmental allergens. Another recent study reported that PFOS exposure decreased baseline airway resistance but significantly increased airway responsiveness in an allergic murine model (Loewen et al. 2011). Furthermore, in our experimental studies, *in vivo* exposure to PFOS was associated with decreased secretion of T-helper (T_H)_1-type cytokines [interleukin (IL)-2 and interferon (IFN)-γ] and increased secretion of T_H_2-type cytokines (IL-4 and IL-10) and IgE, which suggested that PFOS exposure might shift the host’s immune state toward a more T_H_2-like state (Dong et al. 2011). T_H_1/T_H_2 polarization (toward T_H_2 response) is a hallmark of atopy diseases (Colavita et al. 2000), and IgE is known to play a role in mediated type 1 hypersensitivity reactions, including asthma (Platts-Mills 2001). Accordingly, we hypothesized that exposure to PFCs may have a role in asthma development in humans.

Asthma is the most common respiratory disease in young children, and although recent studies indicated that asthma prevalence has plateaued or may be declining (Montefort et al. 2011; Pearce et al. 2007), it is still a major public health problem among young people. The case–control design of the Genetic and Biomarkers study for Childhood Asthma (GBCA) provided an opportunity to explore the association between PFCs exposure and asthma in children. In addition, two validated questionnaires [asthma severity score questionnaire and Asthma Control Test (ACT)] were used to examine the association between PFCs and asthma severity and control in asthmatic children.

## Methods

*Study population.* The GBCA was conducted between 2009 and 2010. A total of 231 10- to 15-year-old children with asthma diagnosed by a physician in the previous year were recruited from two hospitals in northern Taiwan. Controls were selected from our previous cohort study population in seven public schools of Northern Taiwan (Tsai et al. 2010). These schools had diverse geographical and socioeconomic settings, being located in city, rural, and high-altitude communities, respectively. In each targeted school, children of the same age range and without a personal or family history of asthma were invited to participate, and 225 nonasthmatic controls enrolled in the study (response rate was 72% among those contacted by phone). Information pertaining to demographic variables, environmental exposures, and asthma outcomes was collected from questionnaires. We also collected urine and serum samples for each child after 8 hr of fasting. A trained fieldworker measured each child’s height, weight, waist circumference, and blood pressure. All participants and their parents provided written informed consent. The study protocol was approved by the Institutional Review Board (National Taiwan University Hospital Research Ethics Committee) and complied with the principles outlined in the Helsinki Declaration (Declaration of Helsinki 1990).

*Serum IgE, AEC, ECP level detection.* Venous blood was placed into EDTA tubes, a peroxydase coloration was performed, and the absolute eosinophil counts (AEC) were calculated using automatic analyzer (> 10^6^ cells/L; XE2100; Sysmex, Kobe, Japan). Serum samples were stored at –80°C until total IgE and eosinophilic cationic protein (ECP) levels were analyzed. Serum total IgE levels were determined using a Pharmacia UniCap assay test system (Pharmacia Diagnostics, Uppsala, Sweden). Total IgE concentrations < 0.35 IU/mL were defined as absent or undetectable. ELISA kits were used to measure ECP levels in serum samples according to the manufacturer’s instructions (R&D Systems Europe, Abingdon, UK). The limit of quantitation for ECP concentrations was 0.125 µg/mL.

*Asthma control test and asthma severity evaluation.* The ACT, a five-item questionnaire used to assess asthma control in the previous 4 weeks (Nathan et al. 2004), was administered to the asthmatic children. Questions pertaining to asthmatic symptoms, use of rescue medication, and limitation of daily activities are used to ascertain asthma management. The reliability, empirical validity, and discriminative power in assessing the control of asthma by Chinese children are good (Chen et al. 2008). The sum of the scores of the five questions gives the total ACT score (range, 5–25); the higher the score, the better controlled the disease. We also used a 13-item asthma severity score questionnaire to evaluate four overall components of asthma severity in the asthmatic children, including frequency of current asthma symptoms, use of systemic corticosteroids, use of other medications (besides systemic corticosteroids), and history of hospitalizations and intubations (Eisner et al. 1998). Possible total scores range from 0 to 28, with higher scores reflecting more severe asthma.

*PFC concentrations.* PFCs were measured from 0.5 mL of serum using Agilent high-performance liquid chromatography (HPLC)–in tandem with an Agilent 6410 Triple Quadruple (QQQ) mass spectrometer (MS/MS) (Agilent, Palo Alto, CA, USA). Detailed information about standards and reagents, sample preparation and extraction, instrumental analysis, quality assurance and quality control, and recovery experiments in the present study is provided in Supplemental Material, pp. 2–4 (http://dx.doi.org/10.1289/ehp.1205351) and is described elsewhere (Bao et al. 2011). Ten PFCs were analyzed in serum samples: PFOS, PFOA, perfluorobutane sulfonate (PFBS), perfluorodecanoic acid (PFDA), perfluorododecanoic acid (PFDoA), perfluoroheptanoic acid (PFHpA), perﬂuorohexane acid (PFHxA), PFHxS, perﬂuorononanoic acid (PFNA), and perfluorotetradecanoic acid (PFTA). The limit of quantification (LOQ) for PFOS, PFOA, and PFNA was 0.03 ng/mL; for PFBS and PFHxS 0.07 ng/mL; for PFDA and PFDoA 0.1 ng/mL; for PFHpA and PFHxA 0.05 ng/mL; and for PFTA 0.02 ng/mL. All tests were duplicated and average of the two measures was calculated as the concentrations of PFC.

*Statistical analysis.* Statistical analysis was performed using SAS software (version 9.2; SAS Institute Inc., Cary, NC, USA). Concentrations of PFCs and biomarkers below the LOQ were assigned a value equal to the LOQ divided by the square root of 2 for statistical analyses. We calculated univariate statistics, including median, interquartile range (IQR), and range for each PFC. Because PFC concentrations were highly skewed, we utilized the Wilcoxon rank-sum test to compare PFC concentrations between children with and without asthma.

We used logistic regression to estimate odds ratios (ORs) and 95% CIs for each PFC quartile relative to the lowest quartile, with *a priori* adjustment for child age and sex. To determine the magnitude of other potential confounding, we examined the following variables using a backward deletion strategy: parental education, body mass index (BMI), environmental tobacco smoke (ETS) exposure, and month of survey. If the estimated PFC effect changed by at least 10% when a covariate was included in the base model, the covariate was included in the final model. Multiple general linear models were used to estimate associations with continuous outcomes (IgE, AEC, and ECP) in PFC quartiles, with the lowest PFC quartile as reference group and adjusted for identified covariates. These models were applied separately for cases and controls. We modeled an ordinal variable assigned the median value for each corresponding quartile to estimate *p*-values for trend. A *p*-value of < 0.05 was considered statistically significant.

## Results

Compared with children who did not have asthma, asthmatic children tended to be younger and less likely to report ETS exposure (Table 1). In addition, asthmatic children had significantly higher median plasma concentrations of IgE, AEC, and ECP.

**Table 1:** Characteristics of children with and without asthma in the study population.

Characteristic	Without asthma (n = 225)	With asthma (n = 231)	p-Value
Age (years)	13.6 ± 0.7	12.9 ± 1.7	< 0.001
Height (cm)	159.8 ± 7.0	156.6 ± 10.4	< 0.001
Weight (kg)	52.5 ± 13.2	49.8 ± 13.3	0.033
BMI (kg/m2)	20.4 ± 4.1	20.1 ± 3.9	0.379
Sex
Male	102 (45.3)	158 (68.4)	< 0.001
Female	123 (54.7)	73 (31.6)
Parental education
< High school	86 (38.2)	90 (39.0)	0.871
≥ High school	139 (61.8)	141 (61.0)
ETS exposure
No	93 (41.3)	138 (59.7)	< 0.001
Ever	22 (9.8)	23 (10.0)
Current	110 (48.9)	70 (30.3)
Month of survey
July–September	156 (69.3)	106 (45.9)	< 0.001
November–December	69 (30.7)	125 (54.1)
IgE (IU/mL)	331.4 ± 486.6	684.6 ± 679.2	< 0.001
AEC (× 106/L)	152.3 ± 150.3	395.0 ± 280.9	< 0.001
ECP (μg/L)	28.4 ± 41.1	42.2 ± 57.8	0.004
Values are n (%) or mean ± SD.			

Nearly all study participants had detectable serum concentrations of all PFCs (> 94% of PFCs) except for PFDoA (84.4% in children with and without asthma) and PFHpA (53.3% in unasthmatic children, and 70.6% in asthmatic children) (Table 2). Because of the large numbers of samples below the LOQ, we did not conduct further analyses of PFHpA. Serum concentrations of PFCs were significantly higher in asthmatic children than in nonasthmatic children (*p* < 0.05), except for PFHxA concentrations, which were similar in both groups, and PFTA concentrations, which were significantly higher in nonasthmatic children.

**Table 2:** Serum PFC concentrations (ng/mL) in children with and without asthma.

PFC	Without asthma (n = 225)				With asthma (n = 231)				p-Valuea
	Mean ± SD	Median (IQR)	Range	Percent above LOQ	Mean ± SD	Median (IQR)	Range	Percent above LOQ	
PFOS	33.4 ± 26.4	28.9 (14.1, 43.0)	LOQ-148.1	97.3	45.5 ± 37.3	33.9 (19.6, 61.1)	LOQ-149.6	97.0	0.002
PFOA	1.0 ± 1.1	0.5 (0.4, 1.3)	LOQ-11.3	95.1	1.5 ± 1.3	1.2 (0.5, 2.2)	LOQ-9.0	99.6	< 0.001
PFBS	0.5 ± 0.2	0.5 (0.4, 0.5)	LOQ-2.7	94.2	0.5 ± 0.2	0.5 (0.4, 0.6)	LOQ-2.7	99.1	0.022
PFDA	1.0 ± 0.5	1.0 (0.8, 1.2)	LOQ-5.0	95.1	1.2 ± 0.5	1.1 (0.9, 1.5)	LOQ-3.5	97.4	< 0.001
PFDoA	4.5 ± 6.0	2.7 (0.8, 6.0)	LOQ-43.1	84.4	5.8 ± 6.0	3.8 (1.1, 8.4)	LOQ-36.1	84.4	0.014
PFHpA	0.2 ± 0.3	0.2 (LOQ, 0.2)	LOQ-4.3	53.3	0.3 ± 0.5	0.2 (LOQ, 0.3)	LOQ-5.0	70.6	< 0.001
PFHxA	0.2 ± 0.2	0.2 (0.1, 0.3)	LOQ-2.4	98.7	0.3 ± 0.3	0.2 (0.1, 0.3)	LOQ-3.9	97.0	0.765
PFHxS	2.1 ± 2.2	1.3 (0.6, 2.8)	LOQ-11.8	99.6	3.9 ± 9.0	2.5 (1.3, 4.3)	LOQ-129.1	98.3	< 0.001
PFNA	0.9 ± 0.3	0.8 (0.6, 1.1)	0.26–2.5	100.0	1.1 ± 0.5	1.0 (0.7, 1.3)	0.28-3.6	100.0	< 0.001
PFTA	28.9 ± 81.6	5.2 (0.4, 23.3)	LOQ-793.6	99.6	54.6 ± 101.3	4.1 (0.2, 31.7)	LOQ-429.1	99.6	0.003
aWilcoxon rank-sum test to compare the difference of PFC levels between children without asthma and children with asthma.									

Crude and adjusted ORs for asthma in association with the highest versus lowest quartile of exposure were significantly elevated for all PFCs except for PFHxA and PFTA (Table 3). In general, the data suggest increasing odds of asthma with increasing PFCs, with the strongest associations for exposures in the fourth quartile. Specifically, adjusted ORs for the highest versus lowest quartile were 2.63 (95% CI: 1.48, 4.69) for PFOS, 4.05 (95% CI: 2.21, 7.42) for PFOA, 1.90 (95% CI: 1.08, 3.37) for PFBS, 3.22 (95% CI: 1.75, 5.94) for PFDA, 1.81 (95% CI: 1.02, 3.23) for PFDoA, 3.83 (95% CI: 2.11, 6.93) for PFHxS, and 2.56 (95% CI: 1.41, 4.65) for PFNA.

**Table 3:** Association between PFCs and asthma among 456 participants in the genetic and biomarkers study for childhood asthma, Taiwan, 2009–2010.

Exposure		No. of controls	No. of cases	Crude OR (95%CI)	Adjusted OR (95%CI)a
PFOS	Quartile 1b	67	47	1.00	1.00
	Quartile 2	54	60	1.59 (0.94, 2.67)	1.96 (1.11, 3.47)
	Quartile 3	64	50	1.11 (0.66, 1.88)	1.32 (0.75, 2.32)
	Quartile 4	40	74	2.64 (1.54, 4.51)	2.63 (1.48, 4.69)
	p for trendc			0.003	0.003
PFOA	Quartile 1b	71	43	1.00	1.00
	Quartile 2	64	50	1.29 (0.76, 2.19)	1.58 (0.89, 2.80)
	Quartile 3	53	61	1.90 (1.12, 3.22)	2.67 (1.49, 4.79)
	Quartile 4	37	77	3.43 (1.99, 5.93)	4.05 (2.21, 7.42)
	p for trendc			< 0.001	< 0.001
PFBS	Quartile 1b	63	51	1.00	1.00
	Quartile 2	56	58	1.28 (0.76, 2.15)	1.31 (0.74, 2.31)
	Quartile 3	58	56	1.19 (0.71, 2.01)	1.24 (0.70, 2.20)
	Quartile 4	48	66	1.70 (1.01, 2.87)	1.90 (1.08, 3.37)
	p for trendc			0.072	0.021
PFDA	Quartile 1b	70	44	1.00	1.00
	Quartile 2	68	46	1.08 (0.64, 1.83)	1.02 (0.58, 1.80)
	Quartile 3	53	61	1.83 (1.08, 3.10)	1.30 (0.72, 2.33)
	Quartile 4	34	80	3.74 (2.16, 6.49)	3.22 (1.75, 5.94)
	p for trendc			< 0.001	< 0.001
PFDoA	Quartile 1b	60	54	1.00	1.00
	Quartile 2	61	53	0.97 (0.57, 1.62)	0.81 (0.46, 1.43)
	Quartile 3	63	51	0.90 (0.53, 1.52)	0.71 (0.40, 1.26)
	Quartile 4	41	73	1.97 (1.16, 3.36)	1.81 (1.02, 3.23)
	p for trendc			0.021	0.044
PFHxA	Quartile 1b	54	60	1.00	1.00
	Quartile 2	56	58	0.93 (0.55, 1.57)	1.21 (0.69, 2.12)
	Quartile 3	68	46	0.61 (0.36, 1.03)	0.90 (0.51, 1.60)
	Quartile 4	47	67	1.28 (0.76, 2.17)	1.60 (0.90, 2.86)
	p for trendc			0.706	0.168
PFHxS	Quartile 1b	72	42	1.00	1.00
	Quartile 2	69	45	1.12 (0.66, 1.91)	1.54 (0.85, 2.77)
	Quartile 3	45	69	2.63 (1.54, 4.49)	2.94 (1.65, 5.25)
	Quartile 4	39	75	3.30 (1.92, 5.67)	3.83 (2.11, 6.93)
	p for trendc			< 0.001	< 0.001
PFNA	Quartile 1b	69	45	1.00	1.00
	Quartile 2	64	50	1.20 (0.71, 2.03)	1.19 (0.68, 2.09)
	Quartile 3	53	61	1.76 (1.04, 2.99)	1.54 (0.86, 2.76)
	Quartile 4	39	75	2.95 (1.72, 5.06)	2.56 (1.41, 4.65)
	p for trendc			< 0.001	0.001
PFTA	Quartile 1b	52	62	1.00	1.00
	Quartile 2	56	58	0.69 (0.41, 1.16)	0.62 (0.35, 1.09)
	Quartile 3	63	51	0.61 (0.36, 1.02)	0.65 (0.37, 1.14)
	Quartile 4	54	60	0.84 (0.50, 1.41)	0.96 (0.55, 1.67)
	p for trendc			0.410	0.899
aAdjusted for age, sex, BMI, parental education, ETS exposure, and month of survey. bReference category. cp‑Values for exposure were modeled according to the median value of each quartile.					

**Figure 1:**
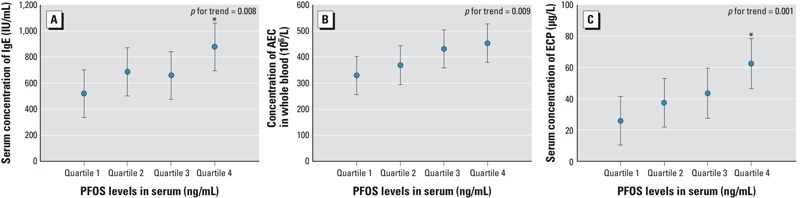
Immunological markers (*A*) IgE, (*B*) AEC, and (*C*) ECP among asthmatic children according to quartiles of PFOS exposure. The data are expressed as estimated mean and 95% CI adjusted for age, sex, BMI, parental education, ETS exposure, and month of survey. *p*-Values for trend were calculated using categories representing the median value of the corresponding quartile (quartile 1: < 19.64 ng/mL; quartile 2: 19.64–33.85 ng/mL; quartile 3: 33.85–61.08 ng/mL; quartile 4: ≥ 61.08 ng/mL). *p < 0.05 compared with quartile 1.

None of the PFCs were significantly associated with serum levels of IgE or AEC among children without asthma, but serum ECP concentration was positively associated with PFDA and PFDoA (Table 4). In contrast, among children with asthma, all three biomarkers were positively associated with PFOS and PFOA, with significant monotonic trends with increasing exposure (Table 5). For example, asthmatic children in the highest of PFOS quartile had mean IgE levels of 877.3 IU/mL (95% CI: 695.2, 1059.5), compared with 517.9 IU/mL (95% CI: 336.7, 699.2) for in the lowest quartile (Figure 1). In addition, with the exception of PFHxA, which was not associated with any of the biomarkers, all the remaining PFCs were associated with two of the three biomarkers evaluated.

**Table 4:** Estimated mean values (95% CI) for serum IgE, AEC, and serum ECP according to serum PFC concentrations among children without asthma (*n* = 225).^*a*^

Exposure		IgE (IU/mL)	AEC (× 106/L)	ECP (μg/L)
PFOS	Quartile 1	286.3 (157.0, 415.6)	138.9 (100.1, 177.8)	29.4 (18.5, 40.3)
	Quartile 2	298.3 (164.6, 432.1)	141.6 (102.2, 181.1)	24.2 (13.0, 35.4)
	Quartile 3	403.5 (274.1, 532.9)	167.8 (128.9, 206.6)	33.5 (22.5, 44.5)
	Quartile 4	336.3 (208.3, 464.2)	160.9 (122.2, 199.7)	26.6 (15.8, 37.4)
	p for trendb	0.404	0.445	0.972
PFOA	Quartile 1	223.1 (76.8, 369.5)	118.5 (78.6, 158.5)	15.4 (3.2, 27.7)
	Quartile 2	298.9 (170.9, 427.0)	110.1 (71.3, 148.8)	28.3 (17.6, 39.0)
	Quartile 3	406.2 (274.6, 537.9)	198.3 (160.7, 235.9)	38.3 (27.3, 49.3)
	Quartile 4	393.9 (258.0, 529.8)	182.0 (139.3, 224.8)	31.2 (19.8, 42.6)
	p for trendb	0.123	0.224	0.133
PFBS	Quartile 1	360.1 (229.6, 490.7)	156.0 (117.6, 194.3)	23.9 (13.0, 34.9)
	Quartile 2	345.0 (214.4, 475.7)	108.2 (70.5, 145.9)	32.1 (21.2, 43.0)
	Quartile 3	329.4 (198.2, 460.6)	151.7 (113.2, 190.3)	28.9 (17.9, 40.0)
	Quartile 4	291.8 (161.9, 421.8)	194.1 (155.6, 232.6)	28.7 (17.7, 39.8)
	p for trendb	0.447	0.070	0.648
PFDA	Quartile 1	248.6 (122.2, 375.0)	118.2 (79.7, 156.7)	18.1 (7.2, 28.9)
	Quartile 2	305.4 (179.9, 430.8)	148.0 (109.7, 186.3)	26.7 (15.8, 37.5)
	Quartile 3	395.6 (267.4, 523.9)	178.7 (140.3, 217.1)	28.1 (17.4, 38.8)
	Quartile 4	379.4 (253.4, 505.5)	164.6 (125.6, 203.6)	40.7 (30.0, 51.4)*
	p for trendb	0.092	0.073	0.004
PFDoA	Quartile 1	358.4 (230.9, 485.8)	89.9 (52.7, 127.1)	19.2 (8.6, 29.7)
	Quartile 2	423.7 (293.2, 554.2)	190.6 (152.4, 228.7)	25.9 (15.1, 36.7)
	Quartile 3	281.8 (151.8, 411.8)	172.0 (134.5, 209.6)	21.7 (11.0, 32.4)
	Quartile 4	261.9 (132.4, 391.4)	158.6 (120.8, 196.4)	46.5 (36.1, 57.0)*
	p for trendb	0.145	0.067	0.001
PFHxA	Quartile 1	215.2 (83.7, 346.7)	127.4 (87.5, 167.4)	25.2 (13.9, 36.5)
	Quartile 2	386.7 (257.7, 515.9)	158.3 (119.4, 197.0)	26.0 (15.1, 37.0)
	Quartile 3	427.9 (299.6, 556.2)	178.7 (140.1, 217.2)	32.1 (21.2, 43.0)
	Quartile 4	296.7 (163.5, 429.9)	144.6 (104.8, 184.3)	30.4 (19.0, 41.7)
	p for trendb	0.330	0.104	0.429
PFHxS	Quartile 1	257.1 (125.3, 389.0)	182.3 (141.9, 222.8)	24.7 (13.4, 36.1)
	Quartile 2	390.6 (259.3, 521.9)	119.3 (80.5, 158.1)	39.6 (28.7, 50.5)
	Quartile 3	363.6 (233.5, 493.8)	147.3 (108.1, 186.4)	25.4 (14.3, 36.5)
	Quartile 4	316.0 (180.4, 451.7)	159.0 (120.0, 198.0)	24.2 (13.2, 35.2)
	p for trendb	0.581	0.321	0.537
PFNA	Quartile 1	278.4 (150.9, 405.9)	138.6 (100.5, 176.7)	28.2 (17.4, 39.0)
	Quartile 2	331.3 (202.8, 459.9)	122.7 (83.7, 161.6)	20.1 (9.2, 31.1)
	Quartile 3	237.5 (108.4, 366.6)	172.2 (134.1, 210.3)	28.7 (17.7, 39.7)
	Quartile 4	474.1 (347.8, 600.4)	175.5 (136.7, 214.3)	36.4 (25.7, 47.1)
	p for trendb	0.084	0.086	0.167
PFTA	Quartile 1	275.6 (142.6, 408.6)	156.7 (117.9, 195.5)	29.7 (18.9, 40.6)
	Quartile 2	330.5 (199.6, 461.5)	133.1 (93.6, 172.6)	34.9 (23.9, 46.0)
	Quartile 3	344.3 (212.6, 476.0)	161.0 (121.8, 200.3)	28.6 (17.6, 39.6)
	Quartile 4	375.0 (245.6, 504.4)	158.5 (118.9, 198.0)	20.4 (9.3, 31.6)
	p for trendb	0.293	0.954	0.196
aModels were adjusted for age, sex, BMI, parental education, ETS exposure, and month of survey. bp‑Values for exposure were modeled according to the median value of each quartile. *p < 0.05 compared with quartile 1.				

**Table 5:** Estimated mean values (95% CI) for serum IgE, AEC, and serum ECP according to serum PFC concentrations among children with asthma (*n* = 231).^*a*^

		IgE (IU/mL)	AEC (× 106/L)	ECP (μg/L)
PFOS	Quartile 1	517.9 (336.7, 699.2)	329.4 (255.8, 403.0)	25.9 (10.4, 41.3)
	Quartile 2	686.2 (501.3, 871.1)	368.6 (293.9, 443.3)	37.4 (21.9, 52.8)
	Quartile 3	658.1 (475.2, 841.1)	431.3 (358.1, 504.6)	43.5 (27.5, 59.4)
	Quartile 4	877.3 (695.2, 1059.5)*	453.4 (379.4, 527.3)	62.4 (46.3, 78.4)*
	p for trendb	0.008	0.009	0.001
PFOA	Quartile 1	512.1 (329.4, 694.8)	325.9 (253.7, 398.1)	30.3 (14.3, 46.3)
	Quartile 2	604.6 (422.1, 787.1)	339.7 (266.8, 412.6)	34.8 (18.9, 50.7)
	Quartile 3	788.2 (607.1, 969.2)	422.1 (349.9, 494.2)	44.3 (28.4, 60.2)
	Quartile 4	836.4 (652.0, 1020.8)*	498.0 (423.7, 572.3)	57.8 (42.2, 73.4)
	p for trendb	0.005	< 0.001	0.010
PFBS	Quartile 1	683.6 (497.0, 870.2)	343.0 (268.8, 417.2)	32.6 (16.3, 48.9)
	Quartile 2	601.2 (416.7, 785.7)	374.0 (301.6, 446.5)	44.8 (29.1, 60.5)
	Quartile 3	671.3 (485.9, 856.8)	380.4 (307.2, 453.5)	42.9 (27.0, 58.8)
	Quartile 4	780.6 (598.4, 962.7)	487.4 (413.4, 561.4)*	47.3 (31.1, 63.6)
	p for trendb	0.496	0.009	0.210
PFDA	Quartile 1	470.6 (289.7, 651.6)	333.6 (256.0, 407.2)	19.0 (3.6, 34.3)
	Quartile 2	615.6 (436.0, 795.2)	351.7 (277.7, 425.8)	45.3 (29.9, 60.7)
	Quartile 3	789.8 (608.2, 971.4)	422.2 (349.3, 495.0)	44.7 (28.9, 60.6)
	Quartile 4	862.2 (682.7, 1041.7)*	470.8 (398.6, 542.9)*	59.7 (44.0, 75.3)*
	p for trendb	0.001	0.004	0.001
PFDoA	Quartile 1	533.0 (348.1, 717.9)	344.1 (270.9, 417.2)	28.7 (13.3, 44.1)
	Quartile 2	653.4 (470.7, 836.0)	385.2 (313.4, 457.0)	36.3 (20.7, 52.0)
	Quartile 3	726.7 (546.6, 906.8)	356.3 (282.9, 429.8)	42.8 (27.3, 58.2)
	Quartile 4	823.5 (640.0, 1006.9)	496.9 (423.8, 570.0)*	62.0 (45.8, 78.2)*
	p for trendb	0.016	0.011	0.003
PFHxA	Quartile 1	539.7 (355.0, 724.4)	397.5 (323.2, 471.7)	36.9 (20.8, 53.0)
	Quartile 2	744.7 (561.1, 928.2)	369.7 (294.7, 444.7)	31.5 (15.3, 47.8)
	Quartile 3	661.7 (480.3, 843.1)	371.9 (298.4, 445.4)	49.5 (33.8, 65.2)
	Quartile 4	794.9 (610.9, 978.8)	443.4 (368.9, 517.9)	49.0 (33.3, 64.6)
	p for trendb	0.075	0.407	0.148
PFHxS	Quartile 1	682.4 (495.0, 869.7)	331.7 (256.3, 407.2)	25.8 (9.5, 42.0)
	Quartile 2	643.0 (456.1, 830.0)	379.1 (305.2, 453.1)	39.6 (24.0, 55.2)
	Quartile 3	679.9 (495.4, 864.5)	430.5 (356.8, 504.2)	41.0 (25.3, 56.6)
	Quartile 4	734.2 (549.1, 919.4)	439.7 (365.5, 513.6)	61.0 (45.4, 76.6)*
	p for trendb	0.632	0.029	0.004
PFNA	Quartile 1	410.9 (230.6, 591.2)	309.7 (236.4, 383.0)	28.8 (13.1, 44.4)
	Quartile 2	704.5 (524.1, 884.9)	353.1 (280.3, 425.8)	34.8 (19.3, 50.3)
	Quartile 3	828.8 (651.6, 1006.0)*	431.7 (359.5, 503.9)	43.5 (27.6, 59.5)
	Quartile 4	790.9 (610.1, 971.6)*	482.5 (411.1, 553.9)*	61.0 (45.3, 76.6)*
	p for trendb	0.001	< 0.001	0.003
PFTA	Quartile 1	541.9 (356.4, 727.5)	328.8 (262.9, 394.8)	36.8 (20.9, 52.8)
	Quartile 2	659.9 (493.4, 826.5)	351.4 (270.5, 432.3)	39.1 (23.3, 54.9)
	Quartile 3	709.6 (507.5, 911.8)	405.0 (332.7, 477.2)	49.9 (31.9, 67.9)
	Quartile 4	833.1 (650.7, 1015.5)	502.3 (429.4, 575.1)*	43.6 (28.9, 58.2)
	p for trendb	0.011	< 0.001	0.409
aModels were adjusted for age, sex, BMI, parental education, ETS exposure, and month of survey. bp‑Values for exposure were modeled according to the median value of each quartile. *p < 0.05 compared with quartile 1.				

Among asthmatic children, positive trends for associations with asthma severity scores were significant for PFOS, PFDA, PFDoA, and PFTA, but none of the PFCs was associated with ACT scores (Table 6).

**Table 6:** Estimated mean score (95% CI) for the relationship between the PFCs levels and asthma severity and ACT among children with asthma (*n* = 231).^*a*^

Exposure		Asthma severity score	ACT score
PFOS	Quartile 1	3.33 (2.36, 4.31)	22.51 (21.71, 23.32)
	Quartile 2	4.18 (3.19, 5.17)	22.72 (21.92, 23.52)
	Quartile 3	4.49 (3.52, 5.45)	22.13 (21.30, 22.94)
	Quartile 4	4.57 (3.61, 5.54)	22.21 (21.41, 23.02)
	p for trendb	0.045	0.450
PFOA	Quartile 1	3.63 (2.66, 4.60)	22.02 (21.22, 22.82)
	Quartile 2	3.99 (3.02, 4.96)	22.14 (21.33, 22.96)
	Quartile 3	4.39 (3.40, 5.38)	22.76 (21.96, 23.56)
	Quartile 4	4.57 (3.59, 5.55)	22.65 (21.84, 23.45)
	p for trendb	0.119	0.168
PFBS	Quartile 1	3.48 (2.50, 4.47)	22.23 (21.41, 23.04)
	Quartile 2	4.42 (3.46, 5.38)	22.39 (21.60, 23.19)
	Quartile 3	3.82 (2.85, 4.79)	22.73 (21.92, 23.53)
	Quartile 4	4.85 (3.88, 5.82)	22.23 (21.42, 23.03)
	p for trendb	0.092	0.836
PFDA	Quartile 1	3.43 (2.48, 4.37)	22.35 (21.54, 23.16)
	Quartile 2	3.79 (2.83, 4.74)	22.55 (21.76, 23.35)
	Quartile 3	4.07 (3.10, 5.04)	22.33 (21.53, 23.13)
	Quartile 4	5.32 (4.36, 6.29)*	22.34 (21.53, 23.15)
	p for trendb	0.005	0.857
PFDoA	Quartile 1	3.68 (2.71, 4.65)	22.57 (21.77, 23.38)
	Quartile 2	3.50 (2.55, 4.45)	22.54 (21.75, 23.33)
	Quartile 3	4.50 (3.53, 5.46)	21.90 (21.10, 22.70)
	Quartile 4	4.91 (3.94, 5.88)	22.57 (21.77, 23.38)
	p for trendb	0.024	0.709
PFHxA	Quartile 1	4.34 (3.36, 5.32)	22.21 (21.40, 23.01)
	Quartile 2	4.10 (3.13, 5.07)	22.41 (21.62, 23.20)
	Quartile 3	4.06 (3.08, 5.04)	21.92 (21.12, 22.72)
	Quartile 4	4.07 (3.09, 5.05)	23.03 (22.23, 23.83)
	p for trendb	0.854	0.284
PFHxS	Quartile 1	3.96 (2.99, 4.94)	21.97 (21.17, 22.78)
	Quartile 2	4.17 (3.19, 5.14)	22.39 (21.59, 23.19)
	Quartile 3	4.44 (3.46, 5.42)	22.48 (21.68, 23.29)
	Quartile 4	4.01 (3.02, 5.00)	22.72 (21.91, 23.54)
	p for trendb	0.722	0.251
PFNA	Quartile 1	4.05 (3.08, 5.02)	22.35 (21.55, 23.15)
	Quartile 2	3.62 (2.64, 4.60)	22.79 (21.98, 23.60)
	Quartile 3	4.25 (3.29, 5.21)	22.09 (21.30, 22.88)
	Quartile 4	4.65 (3.68, 5.63)	22.35 (21.55, 23.16)
	p for trendb	0.217	0.695
PFTA	Quartile 1	3.40 (2.51, 4.28)	22.57 (21.84, 23.30)
	Quartile 2	4.45 (3.39, 5.52)	22.33 (21.44, 23.22)
	Quartile 3	4.05 (3.08, 5.01)	22.01 (21.21, 22.81)
	Quartile 4	4.89 (3.92, 5.86)	22.63 (21.82, 23.44)
	p for trendb	0.050	0.917
aModels were adjusted for age, sex, BMI, parental education, ETS exposure, and month of survey. bp‑Values for exposure were modeled according to the median value of each quartile. *p < 0.05 compared with quartile 1.			

## Discussion

Serum concentrations of PFCs were significantly higher in asthmatic children compared with controls, and among children with asthma, all but one of the PFCs evaluated were positively associated with at least two of the three immunological biomarkers (IgE, AEC, and ECP). Although temporality cannot be determined due to the cross-sectional nature of the data, and noncausal associations due to uncontrolled confounding or other sources of bias cannot be ruled out, the robust associations of PFCs with asthma and asthma related biomarkers in children suggest that a causal relationship may be present. However, concentrations of individual PFCs were positively correlated [see Supplemental Material, Table S1 (http://dx.doi.org/10.1289/ehp.1205351)], and therefore it is not possible to determine whether associations apply to multiple PFCs or to only a subset of individual PFCs.

There is little information in the literature about associations between environmental PFCs and asthma or asthma-related biomarkers in children. In a systematic Medline search (http://www.nlm.nih.gov/bsd/pmresources.html), we identified only two studies of PFCs and atopic disease in humans. The first was a cross-sectional study of 566 residents with prolonged exposure to PFOA in their drinking water (Anderson-Mahoney et al. 2008). In that study, respiratory illness was evaluated by questionnaire, and standardized prevalence ratios (SPR) using National Health and Nutrition Examination Survey (NHANES) data for comparison rates suggested an increased prevalence of asthma among the exposed participants than in the general U.S. population (SPR = 1.82, 95% CI: 1.47, 2.25). The second was a cohort study of prenatal exposure to PFCs in association with IgE levels and atopic dermatitis in 244 newborns (Wang et al. 2011). In that study, prenatal PFOA and PFOS exposures were positively correlated with cord blood IgE levels, but were not significantly associated with atopic dermatitis.

In a murine model of asthma, Fairley et al. (2007) evaluated the effects of PFOA dermal exposure on the hypersensitivity response to ovalbumin (OVA). The authors reported that IgE increased to a greater extent in animals exposed to PFOA and OVA, and that the severity of the OVA-specific airway hyperreactivity response, and a pleiotropic cell response characterized by eosinophilia and mucin production, increased with increasing concentrations of PFOA. Grasty et al. (2005) evaluated the effect of prenatal PFOS exposure on maturation of the terminal airway epithelium in rats based on histological and morphometric examination, and reported that there were significant histologic and morphometric differences between control and PFOS-treated lungs in newborns, suggesting that PFOS may inhibit or delay perinatal lung development. Also, a recent study using an allergic murine model to evaluate effects of PFOS exposure on pulmonary function and airway responsiveness reported that PFOS exposure decreased baseline airway resistance, but significantly increased airway responsiveness in allergic mice (Loewen et al. 2011). In our experimental studies, *in vivo* exposure to PFOS was linked decreased secretion of T_H_1-type cytokines (IL-2 and IFN-γ), and increased secretion of T_H_2-type cytokines (IL-4 and IL-10) and serum IgE, which suggested that PFOS exposure might shift immune responses toward a more T_H_2-like state (Dong et al. 2011). T_H_1/T_H_2 polarization toward T_H_2 responses is a hallmark of atopy diseases (Colavita et al. 2000), and IgE plays a role in mediating type 1 hypersensitivity reactions, including asthma (Platts-Mills 2001). Therefore, we hypothesize that exposure to PFCs may augment the T_H_2 response, resulting in airway hyper-reactivity to environmental allergens.

Potential mechanisms for effects of PFCs on immune response and asthma development in humans are uncertain. One possibility is an effect of PFCs on regulatory T cells that influence the development of immune-related diseases including asthma and allergy (Akbari et al. 2003). Another possible mechanism for effects of PFCs on immune responses pertains to the peroxisome proliferator–activated receptors (PPARs) signaling pathway. PFCs are known as agonists for PPARs (Vanden huevel et al. 2006). Both PPAR-α and PPAR-γ are expressed on cells of the monocyte/macrophage lineage, suggesting a possible role in immune function (Braissant and Wahli 1998). Although PPAR-γ activation has been implicated as an important contributor to the pathogenesis of the toluene diisocyanate–induced asthma phenotype in a female BALB/c mice model (Lee et al. 2006), PFOS and PFOA have been shown to significantly increase activation of mouse or human PPAR-α and PPAR-β, but not of PPAR-γ, *in vitro* (Takacs and Abbott 2007). Lovett-Racke et al. (2004) reported that PPAR-α agonists, including gemﬁbrozil, ciproﬁbrate, and fenoﬁbrate, can increase the production of the T_H_2 cytokine IL-4, and suppress MBP (myelin basic protein) Ac1-11–induced proliferation by T-cell receptor transgenic T cells. In addition, gemﬁbrozil shifted cytokine secretion by inhibiting IFN-γ and promoting IL-4 secretion in human T-cell lines. These results suggest that PFCs may potentially augment the T_H_2 response and subsequent airway hyperreactivity to environmental allergens through a PPAR-mediated mechanism (Fairley et al. 2007).

In the present study, nearly all study participants had detectable serum concentrations of all PFCs, including both asthmatic children and controls. PFOS was the most abundant PFC in the serum measured in 2009–2010 in these Taiwanese children, with median concentrations (28.9 ng/mL in controls and 33.9 ng/mL in asthmatics) that were similar to levels reported for children 12–19 years of age in the general U.S. population in 1999–2000 (29.4 ng/mL), but higher than the median concentration reported for U.S. children in 2007–2008 (11.3 ng/mL) (Kato et al. 2011) and concentrations reported for other populations of children sampled during the mid- to late- 2000s [see Supplemental Material, Table S2 (http://dx.doi.org/10.1289/ehp.1205351)]. In contrast, median concentrations of PFOA in the present study population (0.2 ng/mL in controls and 1.2 ng/mL in asthmatics) were lower than reported for other populations of children during the late 2000s (e.g., 4.0 ng/mL based on NHANES data for 2007–2008), and substantially lower than median concentrations reported for children living in a U.S. community near a manufacturing facility (26.3–32.6 ng/mL) (Frisbee et al. 2010). Differences in PFC concentrations among populations may reflect changes in exposures over time, as well as differences in diet and other sources of exposure and individual differences in rates or patterns of metabolism or excretion.

The limitations of these analyses should be noted. We based the PFCs measures on a single serum sample, and although PFCs have a half-life of 5.4–8.5 years (Olsen et al. 2007), samples taken at several time points might be more accurate than a single sample for classifying exposure. As previously noted, this is a cross-sectional study, and temporal relationships between exposures and outcomes cannot be established. In addition, associations with individual PFCs may be biased due to correlations with other PFCs. Finally, cases were recruited from two hospitals in northern Taiwan, whereas controls were recruited from schools in the same region. Therefore, estimates also may have been influenced by selection bias or uncontrolled confounding.

In conclusion, we observed positive associations between serum PFCs and asthma, and positive associations between PFCs and IgE, AEC, and ECP levels, and (to a lesser extent) asthma severity scores, in asthmatic children. These findings suggest that exposure to PFCs may not be related only to asthma outcomes but also to asthma severity. Although the production of some PFCs has been phased out in the United States and Europe, PFCs are still manufactured in Asia and elsewhere, and exposure may also result from the breakdown of similar compounds to PFCs in the environment (Organisation for Economic Co-operation and Development 2002; U.S. Environmental Protection Agency 2006). Therefore, continued exposure is of public health concern, and additional research on potential immunotoxic effects of PFCs is warranted.

## Correction

In the manuscript originally published online, the unit for IgE was given as IU/dL instead of IU/mL, and the unit for absolute eosinophil count (AEC) was given as 10^9^/L instead of 10^6^/L. They have been corrected here.

## Supplemental Material

(524 KB) PDF
