# Supplementary material for: Serum Polyfluoroalkyl Concentrations, Asthma Outcomes, and Immunological Markers in a Case–Control Study of Taiwanese Children
Source: Environ Health Perspect. 2013 Jan 8;121(4):507–13. doi: 10.1289/ehp.1205351 (PMC3620752; doi:10.1289/ehp.1205351)
Supplement: (524 KB) PDF [file ehp.1205351.s001.pdf]

Supplemental Material

Serum Polyfluoroalkyl Concentrations, Asthma Outcomes, and Immunological Markers in a Case-Control Study of Taiwanese Children

Authors

Guang-Hui Dong, Kuan-Yen Tung, Ching-Hui Tsai, Miao-Miao Liu, Da Wang, Wei Liu, Yi-He Jin, Wu-Shiun Hsieh, Yungling Leo Lee, Pau-Chung Chen

Table of Contents

Standards and Reagents: page 2

Sample Preparation and Extraction: page 2

Instrumental Analysis: page 3

Quality Assurance and Quality Control: page 3-page 4

Supplementary Material, Table S1: page 5

Supplementary Material, Table S2: page 6

References: page 7-page 8

STANDARDS AND REAGENTS

The potassium salt of heptadecafluorooctane sulfonate (PFOS, 98%) was acquired from Fluka (Steinheim, Germany). The potassium salts of perfluorohexane sulfonate (PFHxS, 98%), perfluoroheptanoic acid (PFHpA, 98%), perfluorohexane acid (PFHxA, 98%) were acquired from Interchim (Montlucon, France). The potassium salt of nonafluoro-1-butanesulfonate (PFBS, 98%) and hepatadecafluoropelargonic acid (PFNA, 95%) were acquired from Tokyo Chemical Industry (Tokyo, Japan). Pentadecafluorooctanoic acid (PFOA, 95%) was purchased from Wako Pure Chemical Industries (Osaka, Japan). Nonadecafluorodecanoic acid (PFDA, 96%) and perfluorododecanoic acid (PFDoA, 97%) were purchased from Acros Organics (Geel, Belgium). Perfluorotetradecanoic acid (PFTA, 97%) was purchased from Aldrich (Steinheim, Germany). Tetrabutylammonium hydrogensulfate (TBAHS) of HPLC grade and anhydrous extra pure sodium carbonate (Na_2CO_3, 99.5%) were obtained from Acros Organics (Geel, Belgium). HPLC grade ammonium acetate was obtained from Dikma Technology (Richmond, VA). HPLC grade methyl tert-butyl ether (MTBE), methanol, and acetonitrile were obtained from Tedia (Fairfield, OH). Milli-Q water was cleaned using Waters Oasis HLB Plus (225 mg) cartridges (Milford, MA) to remove the potential residue of PFCs. Mixed stock PFC standard solution was prepared in methanol. All reagents were used as received.

SAMPLE PREPARATION AND EXTRACTION

Serum samples were extracted following a method developed by Hansen et al. (2001) . Two millilitres of 0.25 M Na_2CO_3 and one liter of 0.5 M TBAHS were added to 0.5 mL of serum and then extracted twice with MTBE. The combined MTBE extracts were brought to dryness under a gentle stream of high purity nitrogen, and reconstituted in 1 mL mixture of methanol and 10 mM ammonium acetate (2:3, v/v) before final filtration with a 0.22 μm nylon filter.

INSTRUMENTAL ANALYSIS

Extracts of serum samples were analyzed via high performance liquid chromatography–tandem mass spectrometry (HPLC–MS/MS). Chromatography was performed by an Agilent 1200 HPLC system (Palo Alto, CA). A 25 µL aliquot of extract was injected onto a 2.1 × 100 mm (3.5 µm) Agilent Eclipse Plus C18 column (Palo Alto, CA) with 10 mM ammonium acetate and acetonitrile as mobile phases starting with 40% acetonitrile at a flow rate of 250 µL/min and column temperature of 40 °C. The gradient was increased to 90% acetonitrile at 9 min and then held for 2 min. In addition, an 8 min re-equilibration interval was run before each following sample. The HPLC system was interfaced to an Agilent 6410 Triple Quadrupole (QQQ) mass spectrometer (Santa Clara, CA) operated with electrospray ionization (ESI) in negative mode. Instrumental parameters were optimized to transmit the $[M\text{-}K]^-$ ion before fragmentation to one or more product ions. Declustering potential and collision energies were optimized for each analyte and ranged from 35 to 90V and 10 to 35eV, respectively. Data were acquired by tandem mass spectrometry using multiple reaction monitoring (MRM) at transitions, 499 > 99 for PFOS, 413 > 369 for PFOA, 299 > 99 for PFBS, 399 > 99 for PFHxS, 363 > 319 for PFHpA, 313 > 269 for PFHxA, 463 > 419 for PFNA, 513 > 469 for PFDA, 613 > 569 for PFDoA, 713 > 669 for PFTA. Moreover, multiple daughter ions were monitored for confirmation, but quantitation was based on a single production. In all cases, the capillary was held at -4 kV and the desolvation temperature was kept at 350 °C.

QUALITY ASSURANCE AND QUALITY CONTROL

Procedural blanks were prepared at an interval of every ten samples to check if contamination had occurred during the extraction of samples. Solvent blanks containing acetonitrile and Milli-Q water (2:3, v/v) were run after every twenty samples to monitor for background contamination. Duplicate injections and calibration check standards were run after every twenty samples to assure the precision and accuracy of each run. Matrix spike recoveries were tested by spiking native

standards of all 10 target compounds into 12 randomly selected samples, at levels of 10 ng and 20 ng for each of the target compounds. All the matrix spike samples were analyzed in duplicate. Recoveries of native standards spiked in serum matrix were 98 ± 5%, 101 ± 6%, 95 ± 3%, and 96 ± 5%, for PFOS, PFOA, PFHxS, and PFNA, respectively. The average recoveries for other perfluorochemicals ranged from 82% to 96%. The relative standard deviations (RSD) of duplicate analyses were less than 5% for PFOS, PFOA, PFBS, PFDA, PFHpA, PFHxA, PFHxS, PFNA, PFTA,and less than 10% for PFDoA. The concentrations of serum extracts were quantified via nine-point matrix-matched calibration curves ranging from 0.01 to 100 ng/mL, which were performed by adding mixed PFC standard solution into blank and newborn bovine serum, respectively. The regression coefficients (r^2) of calibration curves for all the target analytes in different matrixes were higher than 0.99. The limit of detection (LOD) was defined as the peak of analyte that needed to yield a signal-to-noise (S/N) ratio of 3:1, and the limit of quantification (LOQ) was defined as the lowest point on the standard curve, above the LOD, with a RSD less than 10%.

Supplemental Material, Table S1. Spearman's rank correlation coefficients (rho) among different PFCs in blood samples (n=456)

PFC	PFOS	PFOA	PFBS	PFDA	PFDoA	PFHpA	PFHxA	PFHxS	PFNA	PFTA
PFOS	1.00	**0.64	**0.27	**0.34	**0.53	**0.27	**0.21	**0.37	**0.35	**0.24
PFOA		1.00	**0.42	**0.41	**0.34	**0.43	**0.26	**0.59	**0.46	*0.11
PFBS			1.00	**0.30	**0.28	**0.29	**0.19	**0.21	**0.18	**0.22
PFDA				1.00	**0.53	**0.31	0.09	**0.42	**0.79	**0.17
PFDoA					1.00	**0.33	*0.10	**0.28	**0.41	**0.71
PFHpA						1.00	**0.29	**0.27	**0.22	**0.24
PFHxA							1.00	**0.33	0.06	0.02
PFHxS								1.00	**0.50	0.05
PFNA									1.00	*0.16
PFTA										1.00

**p<0.01; *p<0.05

Supplemental Material, Table S2. Median serum PFOS and PFOA concentrations (ng/mL) reported for other populations

Reference	Studies/Country	Time	Sample	Age (years)	Median	
					PFOS	PFOA
Kato et al. (2011)	NHANES (USA)	1999-2000	543	12-19	29.4	5.60
	NHANES (USA)	2003-2004	640	12-19	19.9	4.00
	NHANES (USA)	2005-2006	640	12-19	14.9	3.80
	NHANES (USA)	2007-2008	357	12-19	11.3	4.00
Frisbee et al. (2010)	C8 Health Project (USA)	2005-2006	6536	1-11.9	20.7	32.6
	C8 Health Project (USA)	2005-2006	5934	12-17.9	19.3	26.3
OECD (2002)	USA	1995	599	2-12	37.5[a]	----
Holzer et al. (2008)	Siegen, German	2006	80	5-6	5.2[a]	5.2[a]
	Amsberg, German	2006	90	5-6	5.4[a]	24.6[a]
Turgeon et al. (2012)	Canada	2006-2008	86	1-4.5	3.4[a]	1.7[a]
Toms et al. (2009)	Australia	2006-2007	----	6-9	18.3	8.2
			----	9-12	17.7	7.0
Zhang et al. (2010)	China	2009	85	5-10	5.6	2.2
Fei and Olsen (2011)	Danish	1998-2002	787	Pregnancy	34.4	5.4
OECD (2002)	USA	2000	645	20-69	34.9[a]	----
	Sagamihara, Japan	1999	32	Adults	40.3[b]	----
	Tokyo, Japan	1999	30	Adults	52.3[b]	----

[a] Geometric mean; [b] Arithmetic mean;

References

Fei C, Olsen J. 2011. Prenatal exposure to perfluorinated chemicals and behavioral or coordination problems at age 7 years. Environ Health Perspect 119:573-578.

Frisbee SJ, Shankar A, Knox SS, Steenland K, Savitz DA, Fletcher T, et al. 2010. Perfluorooctanoic acid, perfluorooctanesulfonate, and serum lipids in children and adolescents: results from the C8 Health Project. Arch Pediatr Adolesc Med 164:860-869.

Hansen KJ, Clemen LA, Ellefson ME, Johnson HO. 2001. Compound-specific, quantitative characterization of organic fluorochemicals in biological matrices. Environ Sci Technol 35: 766–770.

Haug LS, Thomsen C, Becher G. 2009. Time trends and the influence of age and gender on serum concentrations of perfluorinated compounds in archived human samples. Environ Sci Technol 43: 2131-2136.

Hölzer J, Midasch O, Rauchfuss K, Kraft M, Reupert R, Angerer J, et al. 2008. Biomonitoring of perfluorinated compounds in children and adults exposed to perfluorooctanoate-contaminated drinking water. Environ Health Perspect 116:651-657.

Kato K, Wong L-Y, Jia LT, Kuklenyik Z, Calafat AM. 2011. Trends in exposure to polyfluoroalkyl chemicals in the U.S. population: 1999−2008. Environ Sci Technol 45: 8037-8045.

OECD (Organization for Economic Co-operation and Development) Hazard assessment of perXuorooctane sulfonate (PFOS) and its salts. 2002. Available at: http://www.oecd.org/dataoecd/23/18/2382880.pdf [accessed 17 June 2012]

Toms LM, Calafat AM, Kato K, Thompson J, Harden F, Hobson P, et al. 2009. Polyfluoroalkyl chemicals in pooled blood serum from infants, children, and adults in Australia. Environ Sci Technol 43:4194-4199.

Turgeon O'Brien H, Blanchet R, Gagné D, Lauzière J, Vézina C, Vaissière E, et al. 2012. Exposure to toxic metals and persistent organic pollutants in Inuit children attending childcare centers in Nunavik, Canada. Environ Sci Technol 46:4614-4623.

Zhang T, Wu Q, Sun HW, Zhang XZ, Yun SH, Kannan K. 2010. Perfluorinated compounds in whole blood samples from infants, children, and adults in China. Environ Sci Technol 44:4341-4347.
